# Highly Efficient, Electro-thermal Heater Based on Marangoni-Driven, Oriented Reduced Graphene Oxide/Poly(ether imide) Nanolaminates

**DOI:** 10.1021/acsami.4c17273

**Published:** 2024-12-27

**Authors:** Christos Pavlou, Nikolaos Koutroumanis, Anastasios C. Manikas, Maria Giovanna Pastore Carbone, George Paterakis, Costas Galiotis

**Affiliations:** 1 Institute of Chemical Engineering Sciences, Foundation of Research and Technology- Hellas (FORTH/ICE-HT), Stadiou Street, Platani, Patras 26504, Greece; 2 Department of Chemical Engineering, University of Patras, Patras 26504, Greece; 3 Department of Microelectronics, Faculty of Electrical Engineering, Mathematics and Computer Science, Delft University of Technology, Delft, AA 2600, The Netherlands; 4 Application Driven Research & Innovative Engineering (ADRINE), Patras Science Park, Stadiou Street, Platani, Patras 26504, Greece

**Keywords:** ultrathin rGO films, rGO/PEI nanolaminates, Marangoni self-assembly, high performant heaters, flexible heaters

## Abstract

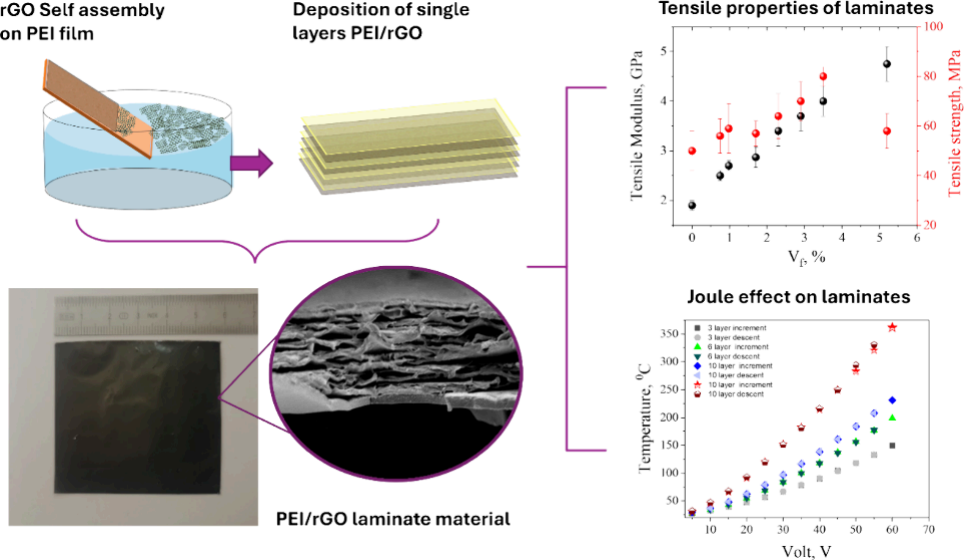

Due to their outstanding electrical and thermal properties, graphene and related materials have been proposed as ideal candidates for the development of lightweight systems for thermoelectric applications. Recently, the nanolaminate architecture that entails alternation of continuous graphene monolayers and ultrathin polymer films has been proposed as an efficient route for the development of composites with impressive physicochemical properties. In this work, we present a novel layer-by-layer approach for the fabrication of highly ordered, flexible, heat-resistant, and electrically conductive freestanding graphene/polymer nanolaminates through alternating Marangoni-driven self-assembly of reduced graphene oxide (rGO) and poly(ether imide) (PEI) films. The microstructure, the mechanical behavior, and the electrical conductivity of the produced Marangoni rGO/PEI nanolaminates are studied as a function of rGO content (up to 5.2 vol %). These nanolaminate thin films show excellent heating properties, with fast heating responses at high temperatures to maximum temperatures at ca. 325 °C due to the Joule heating effect, at maximum rates of 444 °C/s, thus bringing forward an impressive potential of these materials for electrothermal applications. The areal power density was found to be 30 kW/m^2^ for the 5.20% volume fraction of rGO and 325 °C temperature. The robust highly flexible heaters developed in this research hold great promise for a whole range of applications.

## Introduction

Flexible electro-thermal heaters have attracted considerable attention due to their potential in emerging electronic applications ranging from portable and wearable thermal management, de-/ anti-icing, displays, gas sensors, scientific equipment and medical devices.^1−5^ Specifically, regarding thermal applications, the fundamental requirements are rapid thermal response^6−9^ (i.e., quick heating and cooling), wide temperature range of operation with low power consumption, thermal homogeneity over large areas and thermal stability, especially upon mechanical loading. Due to their outstanding electrical and thermal properties, graphene and related materials have been recently proposed as ideal candidates for thermoelectric applications; actually, graphene-based electrothermal heating devices have the advantages of high energy conversion efficiency, fast electrothermal response, and flexibility, and may surpass the current limitation faced using other traditional materials such as metals.^10,11^ Recently many efforts have been made to combine graphene with other materials, such as polymers, to produce nanocomposites, aiming to significantly enhance the physical properties for potential use in high performance applications.^12^ Among them, attempts have been made to develop highly conductive graphene-polymer nanocomposites for heating applications elements, which exhibit also lightweightness, flexibility and resistance to thermal degradation.^11,13^ These materials can be used as heating elements in various applications such as heating pads, clothing, and medical devices. The high thermal conductivity of graphene enables efficient heat transfer and the ability to show rapid heating responses, while the high electrical conductivity enables the use of lower voltage power supplies to achieve the desired heating effect.

However, while these materials offer promising advantages, their long-term stability and durability as heating elements require extensive investigation.^8,14^ All these efforts are mainly based on the use of complex designs or nanocomposites with high graphene contents that may affect the mechanical integrity of the system.^15^ Recently, some of the authors have proposed an alternative approach to produce effective graphene-based composites with fine control of filler distribution, lateral size and orientation, ensuring outstanding properties even at very low graphene content (lower than 1 vol %). The alternation of ultrathin polymer film and large-size monolayer graphene growth via Chemical Vapor Deposition (CVD) in the nanolaminate configuration has been found to preserve the intrinsic physical properties of the continuous graphene sheets leading to superior performance of the graphene-based composites.^16^

In this work we propose a novel nanolaminate architecture, in which thin polymer films are alternated to cm-sized self-assembled ultrathin layers of reduced graphene oxide (rGO). By combining such self-assembly techniques with casting of ultrathin high-performance polymer films, we developed an iterative process for the fabrication of highly oriented, flexible rGO/polymer nanolaminates with several multifunctionalities, surpassing the current limitations faced so far using randomly oriented, discontinuous sheets of graphene. Furthermore, the use of poly(ether imide) (PEI) as polymer matrix has been considered to further exploit the remarkable properties of nanolaminate over a wider range of temperatures, due to high heat and oxidative resistance, chemical inertness, and ability to maintain mechanical and physical properties over a wide temperature range that are typical of high-performance polymers. rGO/PEI with reduced graphene oxide (rGO) content ranging from 0.75 to 5.2 vol % were produced, demonstrating significant improvements in mechanical, electrical, and thermoelectric performance compared to previously reported systems (Table S1). The incorporation of rGO was carefully controlled through a layer-by-layer deposition process, with precise tuning of the volume fractions based on the number of layers. Specifically, systems with 3, 6, and 10 layers of rGO correspond to volume fractions of 0.75%, 1.00%, and 3.5%, respectively. To further explore the potential of this technique, we produced materials with higher rGO content, achieving 1.7% and 5.2% volume fractions by employing double depositions of rGO membranes in 6- and 10-layer systems. Achieving such high rGO volume fractions is a key breakthrough, as conventional techniques such as physical mixing or solution dispersion typically challenge to obtain such levels due to limitations in nanoparticle dispersion, agglomeration, and matrix compatibility. The layer-by-layer approach provides precise control over rGO distribution, enabling higher loadings without compromising homogeneity or causing aggregation. This method significantly enhances electrical and thermoelectric properties by surpassing the rGO percolation network, optimizing electron and phonon transport. These advancements in composite design open new opportunities for high performance applications (e.g., energy harvesting, electrodes, sensors, and flexible electronics).

## Materials and Methods

### Materials

Graphene oxide (GO) was synthesized from large graphite flakes adopting a modified Hummers method.^17^ Preoxidized graphite was produced in the initial step, allowing more effective exfoliation in the second step and production of larger GO flakes. After completion of the reaction and removal of reactants, few-layer GO flakes were separated from aggregates through consecutive centrifugation steps. rGO was finally produced by the reduction of GO by thermal annealing at 1000 °C at inert atmosphere.^18^ The produced rGO was then dispersed in a water/ethanol 60:40 mixture in the concentration of 0.01 mg. The PEI (Extem XH 1015) was kindly supplied by SABIC innovative Plastics. The solutions were prepared by dissolving in cyclohexanone at 80 °C for several hours, at concentrations of 5, 7 and 10%wt.

#### Production of rGO and PEI film

Thin PEI films were fabricated through spin coating of PEI solutions in cyclohexanone on copper foils. Films with different thicknesses were produced by varying the concentration of the solutions and the spinning conditions (from 1000 to 3000 rpm at a 500-rpm interval). After deposition, the film was annealed at 150 °C and subsequently transferred to the Si/SiO_2_ wafer substrate for further characterization. Ultrathin films of rGO were produced using the single-step Marangoni technique, as described in previous work by the authors.^19^ More detailed, 0.01 mg/mL rGO dispersion in water/ethanol has been added dropwise in a deionized water bath and, due to the Marangoni flow, a self-assembly of rGO particles was immediately formed on the water surface. By adopting the scooping technique,^20,21^ the rGO film was then deposited onto a PEI film supported on Cu foil.

#### Atomic Force Microscopy (AFM)

AFM was employed to evaluate the morphology of the produced rGO films and the PEI polymeric membranes. AFM images were acquired by a peak force tapping mode with a Dimension Icon AFM (Bruker Corporation USA). ScanAsyst-Air probes (stiffness 0.2–0.8 N/m, frequency ∼80 kHz) were employed for film morphology and thickness evaluation. The scratch-step method was adopted in the case of PEI films, which were gently scratched with a very sharp scalpel to create a ladder-like step between the surface of the substrate and the nanofilm surface.^22^ The average depth of the scratch below the mean surface plane, corresponding to the film thickness, was measured using the cross-section analysis of the NanoScope Analysis software. In the case of rGO films the thickness was measured by scanning a well-defined step, without any scratch.

#### Raman Spectroscopy

Raman spectra were acquired via a Renishaw Invia Raman Spectrometer with 2400 and 1200 grooves/mm grating equipped with a 785 nm laser line. The laser power on the sample was kept below 1 mW to avoid local heating, while an Olympus MPLN100x objective (NA = 0.90) was used to focus the beam on the samples. Raman mapping was performed on an area of 40 × 20 μm^2^ at a step of 1 μm and 10 s of acquisition time. All Raman peaks were fitted using Lorentzian functions.

#### Mechanical Characterization

The mechanical properties of the specimens were assessed utilizing a microtensile tester equipped with a 5N load cell. The specimens were cut into strips with a length of 35 mm, a gauge length of 25 mm, and a width of 1 mm. The thickness of the specimens was measured with a digital micrometer. The specimens were mounted on paper testing cards and were secured using a cold curing epoxy resin. Subsequently, the specimens were subjected to uniaxial tension at a displacement speed of 0.2 mm/min. The Young’s modulus was estimated via a linear regression analysis of the initial portion of the stress–strain curve. Results for each sample are presented as the average on 10 test specimens.

#### Electrical Characterization

Sheet resistance of the produced rGO layers on PEI film was measured using the van-der-Pauw method;^23^ electrical contacts were formed by pasting copper foil strips to the end of each specimen through a conductive silicone adhesive. A four-point probe positioned on the sample provided the average resistivity under the examined area. Measurements were conducted by using a digital source meter (Keithley 2420). The electrical conductivity σ was estimated by calculating the resistivity  as derived from the resistance values (*R*) shown by the multimeter and the dimensions of the sample.

#### Thermal Characterization

Thermal measurements were performed to assess the Joule heating effect in the produced PEI/rGO laminates. Typical specimens were films of dimensions 40 × 30 mm^2^. Conductive contacts were created on the specimens as described above for the electrical characterization. The PEI/rGO samples were placed on a Teflon holder to guarantee orthogonality with the IR thermal camera while a microthin thermocouple was mechanically attached to the surface of the film. The thermocouple was connected to an USB-4718 8-channel digital input module (Advantech Europe BV, Eindhoven, The Netherlands). The electrodes were connected with a power supply (Keithley 2420 digital multimeter) and the temperature of the specimen was monitored in several points by using the combination of the thermocouple and the Nikon Thermal Vision Camera (3A-SH) with a resolution of 410000 pixels and a 0.1 °C of thermal resolution in the range −20 to 2000 °C). The IR camera, the thermocouple and the power supply were connected with a data acquisition system for collecting the electrical and thermal data of the experiment.

Two-point bending experiments were performed on using a in-house built PTFE stage described elsewhere,^10^ applying compressive displacement to bend the specimen to certain angles in stepwise increments. In these experiments, rectangular specimens having an overall length of 40 mm and a width of 5 mm were mounted on copper frames to ease handling and alignment to the testing machine. Conductive contacts were created on the edges of the specimens with conductive copper tape, and a constant DC voltage of 25 V was applied to the specimen. In order to prevent shortcuts, clamps were coated with dielectric film. The lateral edges of the copper frame were then cut to “free” the specimens before applying the displacement, and the temperature distribution of the specimen was recorded using the thermal IR camera.

#### Scanning Electron Microscopy (SEM)

The cross-section of the produced nanolaminates was examined using field-emission scanning electron microscopy (FESEM, Zeiss SUPRA 35 VP). Each sample was fractured in liquid nitrogen to preserve the structure, followed by gold sputtering to create a conductive coating for imaging

## Results and Discussion

### Production and Characterization of Marangoni RGO/PEI Nanolaminates

**Figure 1:**
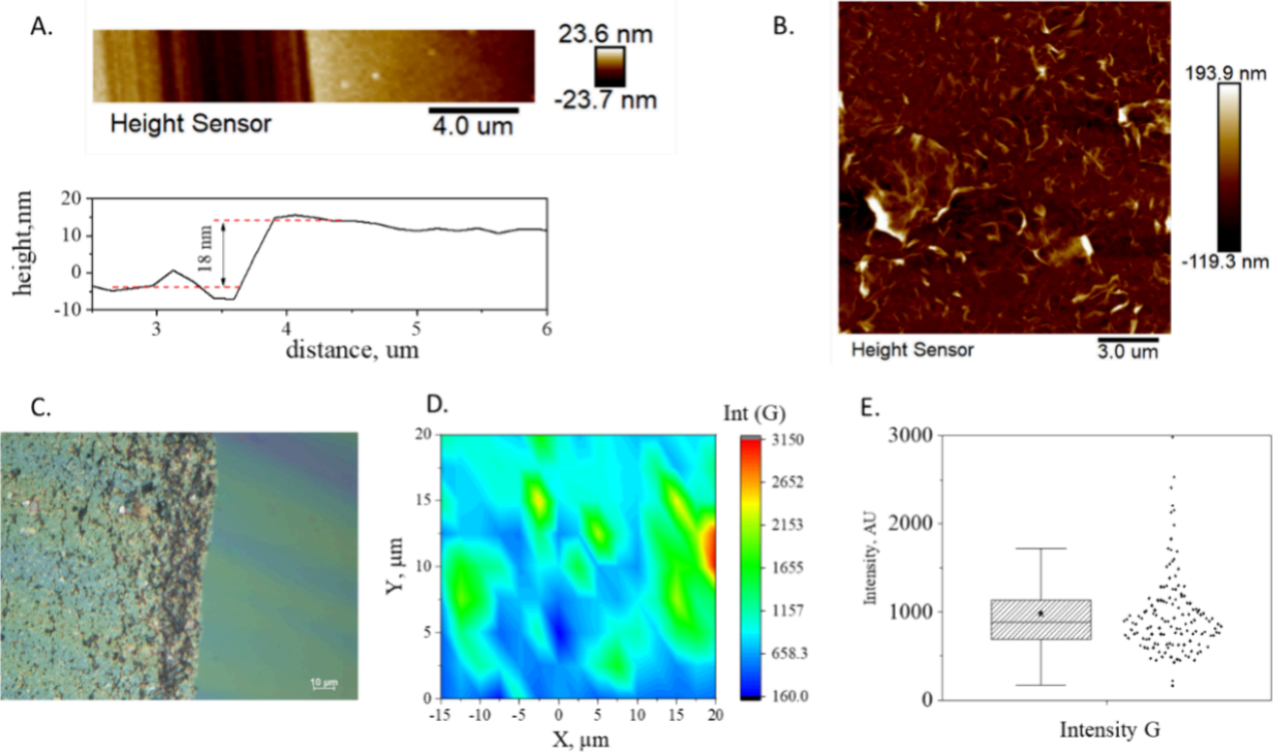
AFM images and height profile (A, B), optical microscopy image (C) and Raman mapping of the intensity of G Peak (D) with the statistical analysis (E) of rGO self-assembly on PEI thin film.

**Figure 2:**
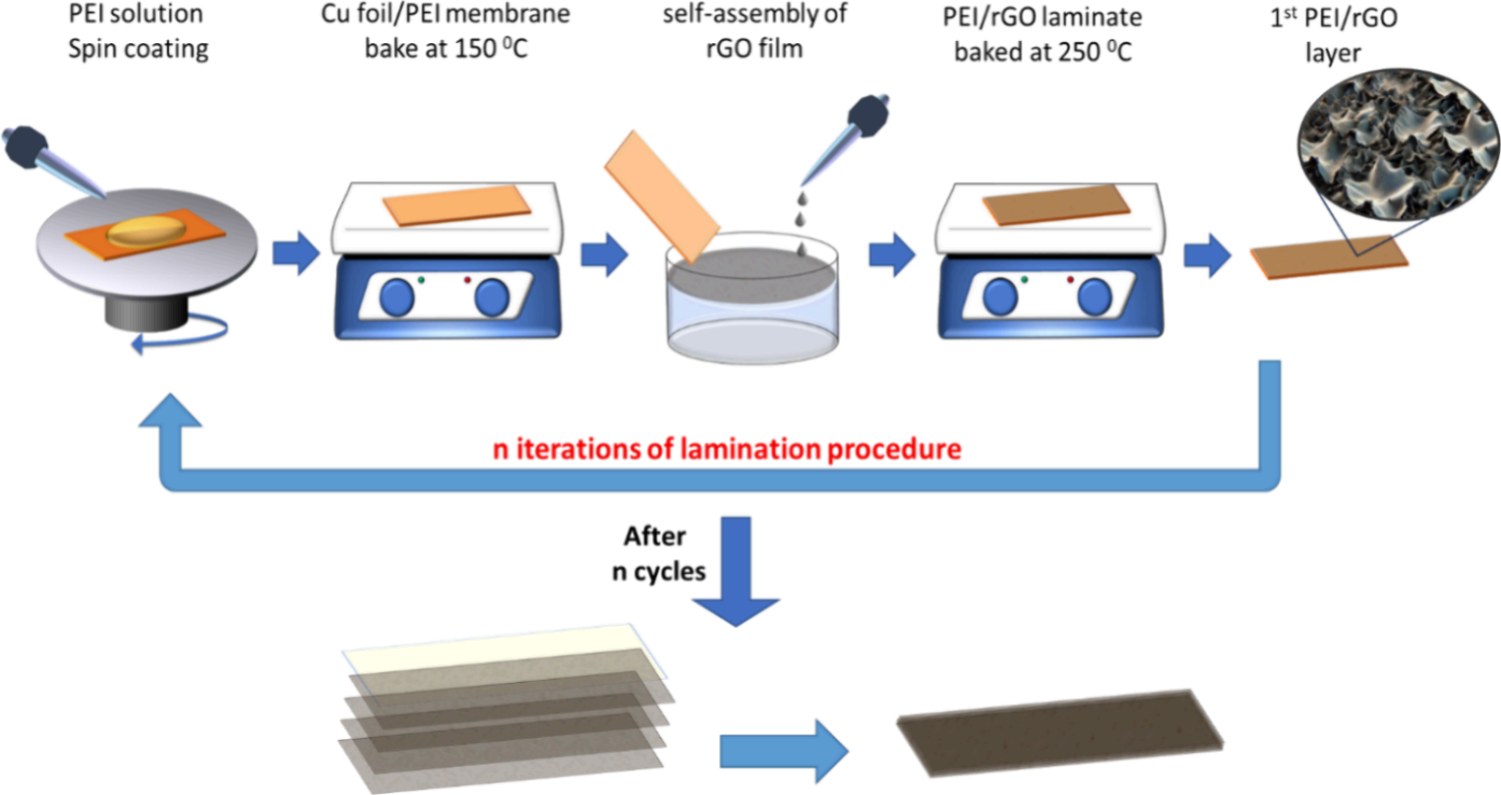
rGO/PEI nanolaminates fabrication process.

As mentioned above, self-assembled ultrathin rGO films were produced through a single-step Marangoni method presented elsewhere.^19^ The morphology and the homogeneity of the produced films deposited on PEI films supported on Si/SiO_2_ wafer were examined by optical microscopy, AFM and Raman spectroscopy. AFM images highlight the characteristic structure of well stacked closely packed rGO flakes and the associated roughness (Figure 1B) but also the thickness of the film (Figure 1A). More specifically, the thickness of the produced rGO films was found to be around 20 nm. The optical observation proves the homogeneity and the continuity of the formed rGO films as it is also presented clearly in Figure 1C. Further proof of the homogeneity of the rGO ultrathin films is provided by Raman spectroscopy. Actually, the homogeneous distribution of the G peak intensity of the mapping area (Figure 1D) and the statistical analysis (Figure 1E) confirm that the formed film has been successfully transferred on the target substrate. Finally, the electrical characterization of the produced rGO films was performed via van the der Pauw method and a sheet resistance of 12 ± 0.5 kΩ/sq was measured. PEI thin films have been prepared through a spin coating process with thickness ranging from 500 nm to 2 μm. The experimental parameters for each thickness are presented in Figure S1. The rGO/PEI nanolaminates were then produced using an iterative layer-by-layer process consisting in the alternation of spin coating of the polymer film on the Marangoni-driven self-assembled rGO layer. The process is presented in Figure 2. The first layer is fabricated by (a) spin coating the PEI solution on a copper foil and (b) annealing above glass transition at 150 °C to remove trapped solvent. Afterward, an ultrathin rGO film (that has been previously formed on a water bath) has been deposited on the top of PEI using the wet transfer technique described elsewhere^16,24,25^ (c). The rGO/PEI/Cu is then annealed at 250 °C to promote adhesion between rGO and PEI layer (d). The steps (a) to (d) are repeated until the desired number of layers has been achieved. Afterward, the sacrificial copper layer is etched away using a 0.15 M Ammonium Persulfate (APS) solution, releasing a freestanding nanolaminate Marangoni rGO/PEI film. Furthermore, a PEI reference sample was prepared according to the procedure described in.^26^

**Figure 3:**
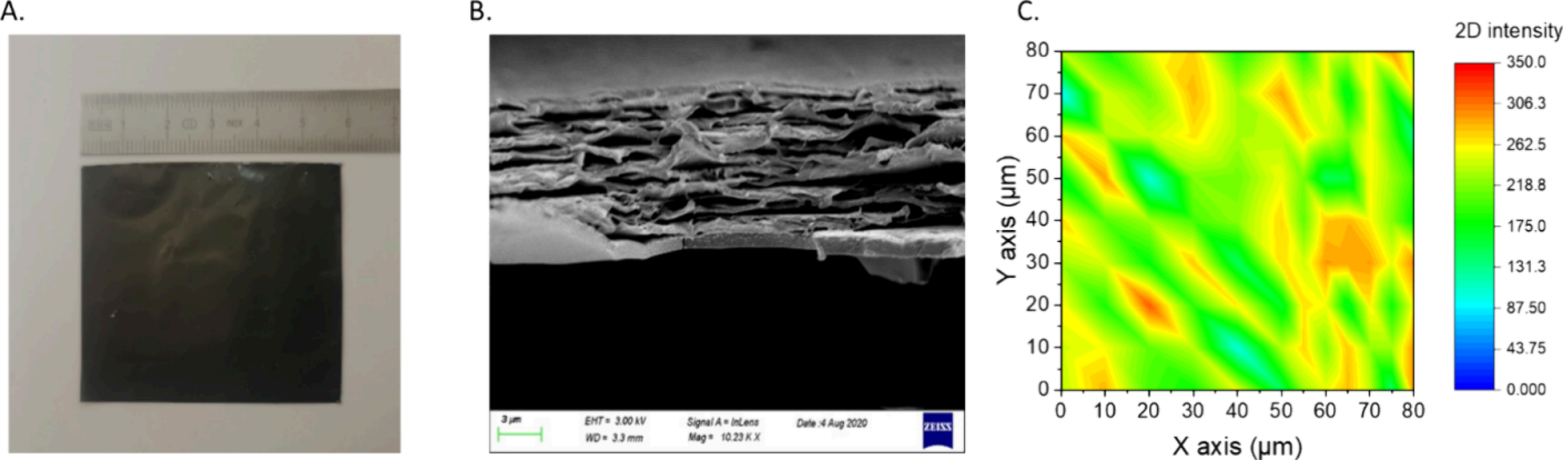
Centimeter-sized rGO/PEI nanolaminate (A), SEM image of the cross section of the samples highlighted the laminate structure (B) and Raman mapping G peak intensity highlighted the uniform distribution (C).

As for similar nanolaminate architectures,^16,27^ the repetitive unit cell in the Marangoni nanolaminate is a ply consisting of a rGO self-assembled layer and a PEI film, therefore the volume fraction of the filler can be defined as
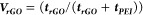
1where ***t**_**rGO**_* is the thickness of the rGO self-assembly (which is kept constant in all the experiments, and equal to 20 nm) and ***t**_**PEI**_* is the thickness of the single PEI layer. rGO/PEI nanolaminates with rGO content ranging from 0.75%vol to 3.5%vol were produced and this was achieved by modulating the thickness of the polymeric film produced via spin coating, with ***t**_**PEI**_* ranging from 500 nm to 2 μm. In each experiment, the concentration of the PEI solution and the spinning conditions were adequately adjusted to obtain the desired polymer film according to the preliminary thickness evaluation process. Ιn addition, laminates with higher rGO content (up to 5.2%) were produced by introducing a double deposition of rGO self-assembly for each PEI layer, thus leading to a [PEI/(rGO)_2_] repetitive unit. It is important to note here that, for each nanolaminate, the number of plies and therefore the final thickness (around 5 μm) was determined in order to guarantee a safe handling of the produced membranes during characterization. The information on the spin coating process employed to produce the PEI films, along with the correspondence between the volume fraction of rGO in the nanolaminate and the thickness of the polymer in the [rGO/PEI] or the [PEI/(rGO)_2_] ply, is summarized in Figure S1. Finally, cm-sized nanolaminates were fabricated as shown in the picture depicted in Figure 3a. SEM image of the cross section of the produced films reveals the laminate architecture of the produced composites (Figure 3b). The homogeneity of the produced nanolaminates was verified also from the uniform spatial distribution of intensity of the G peak presented in the Raman mapping (Figure 3c) confirming that the rGO self-assembly is successfully transferred in each iteration on the thin polymeric layer.

### Mechanical Characterization

**Figure 4:**
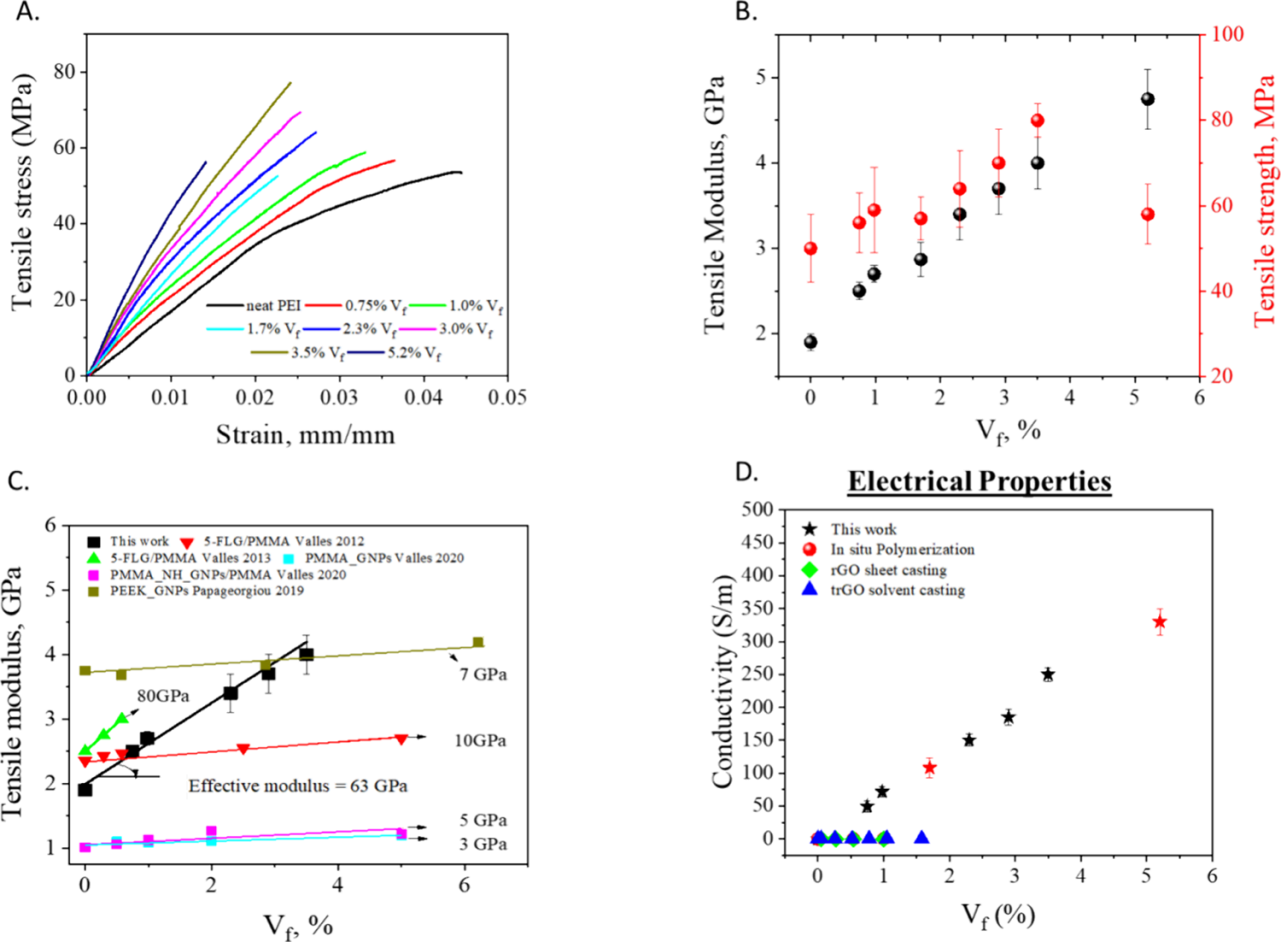
Stress–strain curves (A), tensile modulus and tensile strength (B), effective tensile modulus compared to the state of the art (C) and electrical properties (D) of rGO/PEI nanolaminates for the different volume fractions of rGO.

The mechanical properties of produced rGO/PEI nanolaminates were assessed by uniaxial tensile loading. Representative stress–strain curves for neat PEI and rGO/PEI nanolaminates with different rGO volume fractions are depicted in Figure 4a, highlighting a significant improvement in both Young’s modulus and tensile strength, which is however accompanied by a gradual reduction of strain at break with the increase of rGO content (Figure S2). Further stiffening is observed for the [PEI/(rGO)2] laminate with the highest rGO content (5.2%), albeit a significant embrittlement of the material and reduction of strength are observed. Young’s modulus estimated via a linear regression analysis of the initial portion of the stress–strain curve and the ultimate tensile strength are reported as a function of rGO volume fraction in Figures 4b, highlighting a linear dependence for all the laminates. The maximum improvement of both tensile strength and modulus was observed in the nanolaminate with 3.5%vol of rGO, with an increment of 60 and 110%, respectively, compared to the neat PEI. This is significantly higher than previously obtained in PEI filled with functionalized graphene in a discontinuous composite architecture.^28^ As proposed earlier by some of the authors,^19^ by using a simple rule of mixture the effective contribution of rGO to the modulus and the strength of the nanolaminate can be estimated, yielding 63 GPa and 892 MPa, respectively. These values are quite high and not previously observed for graphene flakes for which their small lateral dimensions (usually 1–5 μm) are not sufficient for effective stress transfer.^29,30^ In contrast, the Marangoni technique that is based on an oriented fish-scale morphology, as demonstrated also in Figure 2, allows a more efficient stress transfer from the polymer to the inclusion under axial deformation.

The produced rGO/PEI nanolaminates were characterized also in terms of in-plane electrical conductivity and results are plotted in Figure 4d as a function of rGO volume fraction. A linear dependence on rGO content reveals that the layer-by-layer assembly has no percolative behavior of the electrical in-plane conduction, as already observed for similar architectures.^16,31^ This is further confirmed by the fact that the linearity is still maintained in [PEI/(rGO)2] laminates, which in fact present a double rGO layer in each repetitive unit. In particular, the electrical conductivity increases linearly up to 330 S/m at the highest rGO content.

The linear regression analysis of the curve provides an estimation of the effective conductivity of the Marangoni rGO layer, which has been found close to 6000 S/m. Due to the highly aligned and close-packed rGO flakes achieved through the innovative approach based on Marangoni-driven self-assembly, the percolative conduction mechanism is undetectable as conduction bridges are formed at extremely low rGO content thus giving the possibility to achieve conductivity values never achieved before.^32−34^ Apparently the percolative conduction mechanism remains present, its direct observation is hindered due to the distinct morphology shaped by the Marangoni process. The self-assembly of rGO flakes rapidly forms continuous conductive networks during the early stages of deposition, minimizing dependence on the conventional percolation threshold typically observed in less-ordered systems. In conventional composites, percolation requires a critical volume of conductive filler to form interconnected pathways. However, in this case, the precise alignment and compact packing of rGO flakes ensure conductive paths emerge at significantly lower concentrations, effectively concealing typical percolative behavior. This unique structural organization shifts the conduction properties from random filler dispersion to ordered networks, resulting in enhanced conductivity before the system reaches classical percolation thresholds.

#### Thermal Characterization

The electrothermal performance of rGO/PEI nanolaminates, driven by the improved electrical properties from increased rGO content and the thermal and oxidative stability of PEI, has been evaluated for their application in Joule heating. This process, for which electrical energy is converted into heat, benefits from the unique characteristics of carbon nanomaterials such as high surface area-to-volume ratio, superior thermal conductivity, and distinctive structure, despite their higher electrical resistivity compared to metals.^10^ Carbon nanomaterials, including graphene and CNTs, demonstrate quick thermal response to voltage due to efficient heat dissipation and transfer, aided by their material structure and electron–phonon coupling. These properties make them superior for rapid thermal applications, guiding the optimization of rGO/PEI nanolaminates for enhanced electrothermal uses. This approach herein is aimed at dissecting the Joule heating effect in electrothermal materials, where electrical energy is converted to heat, as dictated by Joule’s law through the equations *P = I*^*2*^*R* and *P = V*^*2*^*/R*. Resistance *R*, influenced by the material’s resistivity ρ and geometry, *R* = ρ*l/A*, and its inverse relationship with electrical conductivity, ρ = *1/*σ, underlines the efficiency of heat generation: materials with high electrical conductivity exhibit lower resistivity, facilitating efficient heat production under identical electrical loads. This principle is essential for the ability of the produced nanolaminates to swiftly generate and modulate heat, crucial for their application in various electrothermal domains. Our experiments target the evaluation of these nanolaminates as superior electrothermal materials, leveraging their electrical and thermal conductivities (σ and *k* respectively) to ensure efficient, controlled heating. High electrical conductivity is expected to promote efficient current flow with reduced resistive heating, while increased rGO content should enhance thermal conductivity, optimizing heat distribution and dissipation.

**Figure 5:**
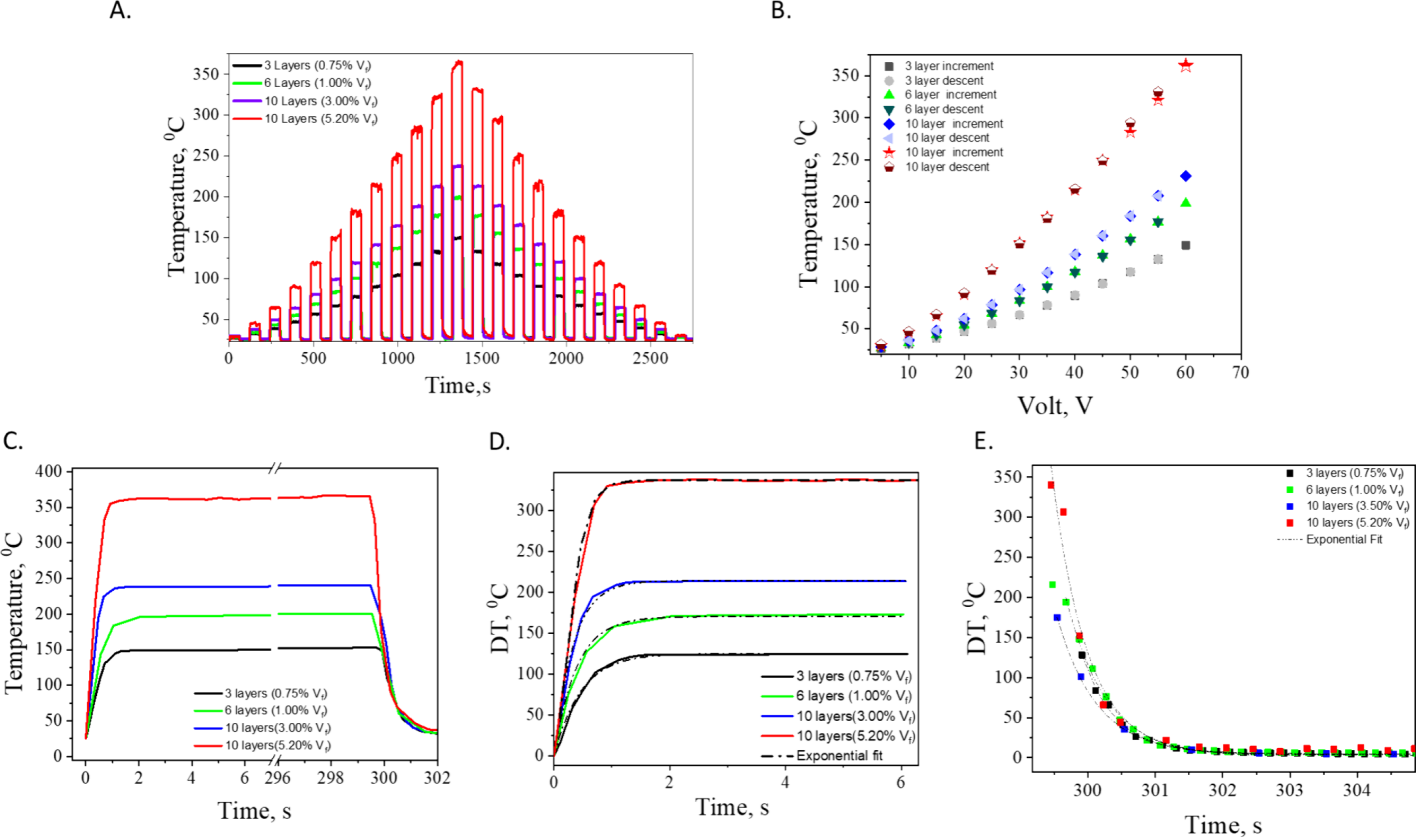
Temperature response of rGO/PEI nanolaminates under control electrical stimulus (A), Temperature response under increment and decrement under systematically and decreased with steps of 5 V (B), temperature profiles of the PEI/rGO nanolaminates, under a constant electric voltage of 60 V applied over a 300-s interval (C) and focused on the increment and decrement time zones (D, E).

In Figure 5a it is revealed the temperature response of the rGO/PEI nanolaminates under controlled electrical stimulus. The temperature of the nanolaminates was recorded by incrementing the DC electrical potential in steps of 5 V, beginning at 5 V and escalating up to 60 V. This stepwise modulation allowed for a nuanced observation of the Joule heating effect across rGO concentrations of 0.75%vol, 1.00%vol, 3.50%vol and 5.2%vol within the nanolaminates. As the applied voltage increased, a corresponding intensification in temperature was observed, showcasing the capability of the nanolaminates to efficiently convert electrical energy into thermal energy. The maximum steady-state temperatures achieved by the nanolaminates were dependent on the rGO content, with temperatures ranging from 120 to 345 °C at the apex of the applied potential. This range highlights the influence of laminate configuration and graphene content as well, on the thermal performance of the materials. The figure presents a clear, tree-like profile of temperature responses over time, exhibiting a stable and repeatable heating behavior devoid of any hysteresis. The observed uniformity in temperature distribution (<2%) derived from a detailed analysis of the thermal image depicted in Figure S3, highlights the consistency in heating across the sample. This uniformity, along with the controlled heating rates, further underscores the suitability of rGO/PEI nanolaminates for advanced applications where thermal management is critical (see Figure S3).

In Figure 5b an extended analysis of Figure 5a is presented, which delineates the maximum steady-state temperatures achieved by PEI/rGO nanolaminates at varying rGO concentrations as voltage is incremented and decremented, revealing a stable consistency in thermal response. As the applied voltage is systematically increased in 5 V steps intervals up to 60 V and then decreased back to 5 V, the temperatures attained at corresponding voltages remain consistent across the cycles. This uniformity suggests that the materials exhibit negligible thermal hysteresis and do not retain excess heat that could impact the system’s heat capacity in a significant manner. It is important to acknowledge that for the 10-layer nanolaminate with 5.20% rGO, minor disparities in temperature were noted at points exceeding 270 °C. However, these variations are not substantial enough to strongly support the presence of significant thermal lag or hysteresis within the material. This is particularly noteworthy given that the experiments were carried out under ambient conditions. The slight differences observed at these higher temperatures could be attributed to normal experimental variances as monitored by the thermocouple rather than a pronounced material characteristic. These findings of Figure 5a-b suggest that PEI/rGO nanolaminates, across the examined rGO concentrations, possess stable and predictable electrothermal properties. This stability is crucial for their application in technologies that require precise thermal control. The minimal hysteresis observed, even at higher temperatures, accentuates the potential of these materials for reliable use in practical environments, where conditions are often far from ideal. The obtained data provides a solid foundation for the subsequent cyclic stability tests, which aim to further investigate the resilience and durability of the thermal performance of the nanolaminates under repetitive heating and cooling conditions below 250 °C. Furthermore, a detailed analysis of the temperature profiles over time and applied voltage was carried out to quantify the heating and cooling rates of the samples. The detailed temperature profiles of the PEI/rGO nanolaminates, captured under a constant electric voltage of 60 V applied over a 300-s interval, are depicted in Figures 5c. An insight is provided into the thermoelectric behavior of the nanolaminates, revealing a rapid temperature increase to a maximum steady-state condition that is maintained until the external electric potential is removed. Upon removal of the voltage, a quick temperature descent occurs, demonstrating the capacity of the material for swift thermal regulation. Specifically, the 0.75%vol and 1.0%vol rGO samples demonstrate distinct temperature profiles, with the 0.75%vol rGO nanolaminate reaching a steady-state temperature of approximately 127 °C at a rate of 145 °C/s. In comparison, the 1.0%vol rGO nanolaminate achieves a higher steady-state temperature of approximately 175 °C at a rate of 180 °C/s. Both specimens exhibit the capability to attain their maximum temperatures within roughly one second, highlighting their potential for rapid heating applications. The 3.00% rGO nanolaminate stands out with a notable heating rate of 330 °C/s, reaching a steady-state temperature of about 240 °C. This pronounced heating rate, along with the higher temperature threshold achieved, suggests that even a moderate increase in rGO content significantly enhances the electrothermal performance of the nanolaminates. This trend is consistent with the expected impact of rGO concentration on the electrical and thermal conductivities of the nanolaminates, which in turn influence the heating rate. For the 5.20% rGO content sample, a significant heating rate is evident, with the temperature reaching approximately 360 °C at a rate of 444 °C/s. This rapid heating is followed by an equally swift cooling phase once the voltage is removed, indicating the nanolaminate’s ability to achieve and shed high temperatures in short order. Samples with lower rGO content display varying maximum steady-state temperatures and rates of heating, indicating that the rGO concentration influences the electrothermal properties of the nanolaminates. The collective data from the nanolaminates with various rGO contents demonstrates a clear correlation between rGO volume fraction and both the steady-state temperature and the rate of heating. The ability of these nanolaminates to reach their maximum temperatures so rapidly underlines the efficiency of the Joule heating mechanism within these materials, as well as their suitability for applications that necessitate quick thermal transitions. The comprehensive analysis of the temperature profiles (Figure 5a, along with Figures S4 and S5) offers critical insights into the heating and cooling dynamics of PEI/rGO nanolaminates, paving the way for their design optimization in precise thermal management applications. In Figures 5d-e a detailed analysis of the heating and cooling rates for PEI/rGO nanolaminates when subjected to a maximum applied voltage of 60 V is shown. These figures enable the estimation of the characteristic time growth constant τ_*g*_ and the decay time constant τ_*d*_, which are indicative of the material’s response times to thermal stimuli. τ_*g*_ is defined as the time required for the temperature to rise from the initial temperature ***T**_**RT**_* to a certain percentage of the maximum steady-state temperature ***T**_**smax**_*, as described by the equation:^4,10^
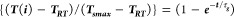
2

Conversely, τ_*d*_ quantifies the cooling rate, representing the time taken for the temperature to decrease from ***T**_**smax**_* to ***T**_**RT**_* following the empirical equation:^4,35^
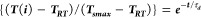
3where the ***T***(***i***) is the random temperature at time τ_**g**_. As shown in Figure 5d, e by fitting the temperature profiles to eq 2 and 3, the values of τ_**g**_ and τ_**d**_ parameters have been estimated and are listed in Table 1, for a given 60 V of DC potential. The characteristic growth time τ_***g***_ is found to decrease with rGO content while the characteristic decay time τ_***d***_ does not vary significant thus considering a stable behavior independent to the rGO content.

**Table 1:** Increment Time (τ_g_) and Decrement Time (τ_d_) of Temperature At Different RGO Volume Fraction

**rGO V**_**f**_	τ_**g**_	τ_**d**_
**0.75%**	0.46	0.52
**1.00%**	0.38	0.56
**3.00%**	0.33	0.53
**5.00%**	0.2	0.55

The decrease in τ_***g***_ with an increase in rGO content reveals a significant aspect of their thermal response: higher rGO percentages allow for a faster attainment of maximum temperatures. This enhanced rate of temperature increase is directly linked to the improved electrical and thermal conductivities offered by greater rGO content, which accelerates the Joule heating process. On the other hand, the decay time constant τ_***d***_, which measures the rate of cooling, exhibits a nonvariable behavior upon rGO concentration. This suggests that the cooling rate is less influenced by the amount of rGO and more dependent on the inherent characteristics of the nanolaminates, such as their dimensions, weight, and overall material structure. This observation of consistently low τ_***d***_ values across all rGO concentrations underscores the adeptness of nanolaminates in thermal regulation, showcasing their ability to dissipate heat quickly and return to ambient conditions postvoltage application. Such a trait is particularly advantageous for technological applications that require quick thermal adaptability. In devices like thermal switches, where the transition between conductive and insulative states must be prompt, or in applications involving temperature-regulated surfaces that demand a rapid thermal response, the PEI/rGO nanolaminates’ capacity for fast and controlled heating and cooling is invaluable. These properties not only enhance the versatility of these materials but also broaden their applicability in advanced thermal management systems.

**Figure 6:**
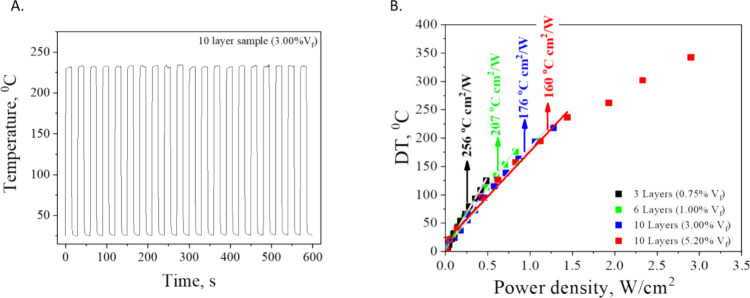
Cyclic test on 10 layers rGO/PEI nanolaminates (A), power density for different rGO/PEI nanolaminates volume fractions for each applied electrical potential (B).

In Figure 6a the results from cyclic tests conducted on a 10-layer PEI/rGO nanolaminate with a 3.00% rGO volume fraction are presented. The tests were performed by applying a 40-V on–off voltage cycle with 15-s intervals over a total duration of 600 s. The nanolaminate consistently reached a maximum temperature of approximately 240 °C, exhibiting no significant temperature fluctuations during the steady-state heating phase. This behavior indicates a robust thermal equilibrium within the nanolaminate structure, able to quickly reach and maintain a stable temperature with each applied voltage cycle. The stability demonstrated by these cyclic tests suggests that the nanolaminate possesses ideal electrothermal properties, including the ability to equilibrate and dissipate residual heat effectively within a period of 15 s. Such a characteristic is indicative of the nanolaminate’s capability to handle rapid heating and cooling transitions without experiencing detrimental thermal effects. The consistent attainment of a steady maximum temperature further implies the absence of thermal degradation or performance loss over the course of the testing period.

Very interestingly, as shown in Figure S6, the thermoelectric behavior of the nanolaminates is retained even under bending, with a homogeneous temperature throughout the specimen; moreover, the heating performance is not significantly affected by the degree of bending thus suggesting the great potential of the proposed nanolaminates for flexible electronics applications and even wearable devices, which need to flex during use. The observed thermal performance paves the way for deploying these materials in systems where temperature control and stability are crucial.

Finally, the thermal resistance efficiency of PEI/rGO nanolaminates has been assessed by conducting a linear regression analysis on the experimental data, which correlates the maximum steady-state temperature rise (Δ*T*) with the power density (P) for each applied DC electrical potential.^36,37^Figure 6b presents these findings, displaying the calculated thermal resistance values that range between 150 to 256 °C·cm^2^/W. This range indicates a substantial variance in heat retention capabilities and energy consumption efficiency across the nanolaminates with different rGO contents. The nanolaminates with a 0.75% rGO volume fraction exhibit the highest thermal resistance efficiency at 256 °C·cm^2^/W, suggesting a greater resistance to heat flow and an enhanced capacity to retain heat for a given amount of power input per unit area. In contrast, nanolaminates with increased rGO content show lower thermal resistance efficiency values at 207 °C·cm^2^/W for 1.00% rGO and 173 °C·cm^2^/W for 3% rGO indicating a better ability to conduct heat away from the source. Notably, the 5.20% rGO content sample stands apart with a thermal resistance efficiency of 150 °C·cm^2^/W, the lowest among the samples tested. This suggests that with a higher rGO content, the ability of the material to conduct heat is enhanced, leading to a more efficient heat transfer and a lower temperature rise per unit of power density. These higher values point to an increased resistance to heat flow and a heightened ability to retain heat within the material for a given power input. This behavior is particularly advantageous in applications that require thermal insulation or where it is essential to maintain a temperature differential across the material. The observed decrease in thermal resistance efficiency with increasing rGO content can be partially attributed to the reduction in interlayer thickness of the PEI matrix.^37^ As the volume fraction of rGO increases, the distance between the polymer layers decreases, which can have a significant impact on the thermal conductivity, particularly in the cross-plane direction of the nanolaminates. A thinner polymer matrix facilitates better heat conduction through the material, leading to a more rapid heat dissipation and a lower thermal resistance efficiency.^36,37^

Finally, Table S1 presents a comprehensive comparison with similar studies, highlighting the uniqueness of our results. More detailed, their enhanced flexibility that allows the heater to conform to irregular surfaces and be used in applications where rigidity is a limitation; the fast heating and response times due to the reduced thermal mass of the thin polymer films; their lightweight nature that make them suitable for portable or wearable applications; and their ability to tailor the polymer matrix, such as using graphene or rGO, can enhance thermal conductivity, potentially improving heating efficiency and power consumption when compared to standard metal or ITO-based heaters. Only systems incorporating fibers or metal particles are known to reach significant heating rates and temperatures exceeding 300 °C. This work represents a notable advancement in the field, demonstrating performance metrics that surpass these benchmarks for flexible membranes.

## Conclusions

In this work it is presented a novel and a facile fabrication technique for the production of graphene-polymer nanolaminates which is based on the subsequent assembly of alternating layers of graphene and a high-performance polymer matrix aiming to produce stiff and electrically conductive large-sized rGO/PEI laminates capable to present multifunctional capabilities such as the Joule heat effect. By adopting this approach, we achieved precise control over the mechanical and electrical characteristics of the laminates leading to multifunctional behavior. In terms of mechanical properties, the produced freestanding PEI/rGO laminates showed significant improvement, with a 2-fold increase in modulus of elasticity, reaching up to ca. 4.75 GPa. Linear regression analysis estimated the effective modulus of the rGO layers at 63 GPa, demonstrating the effectiveness of our assembly process in enhancing material performance. Triggered by high electrical conductivities the laminates displayed high conductivity, up to 330 S/m, placing them as ideal candidates for heating elements through the Joule heating effect. The heating resistors were capable of achieving maximum temperatures of 360 °C in less than one second. Notably, when voltages exceeding 20 V are applied, the high graphene volume heater can rapidly reach temperatures above 80 °C in subsecond time, achieving impressive heating rates of up to 444 °C/sec. Beyond their electrothermal behavior, the rGO/PEI laminates exhibit a thermally stable behavior and obtain high thermal resistances which can be up to 256 °C cm2/W. This fast and stable heating behavior, coupled with exceptional heat generation and thermal stability, makes these laminates ideal for applications requiring precise, rapid thermal control-such as deicing systems, wearable heaters, and medical devices; while the robust properties of the PEI matrix ensure suitability for harsh environments where both durability and efficient heat management are critical.

## Supporting Information Available

Figure S1: Thickness of PEI nanofilms as a function of angular speed for polymer solutions with different PEI concentrations in cyclohexanone; Figure S2: Strain at break values of rGO/PEI nanolaminates for the different volume fractions of rGO; Figure S3: Cyclic test on 10 layers double rGO/PEI nanolaminates and a thermal image of mounted sample at 40 V; Figure S4: Temperature increment versus time of rGO/PEI nanolaminate with 5.20% volume, for maximum applied voltage; Figure S5: Measured heating rates of rGO/PEI nanolaminates for the different volume fractions of rGO; Figure S6: Real-time IR images of the 10-layer nanolaminate with *V*_f_ = 5.2% upon incremental bending; A DC voltage of 25 V is applied to the specimen; Table S1: Joule heat benchmarking (PDF)
