# Supplementary material for: Highly Efficient, Electro-thermal Heater Based on Marangoni-Driven, Oriented Reduced Graphene Oxide/Poly(ether imide) Nanolaminates
Source: ACS Appl Mater Interfaces. 2024 Dec 27;17(1):2000–9. doi: 10.1021/acsami.4c17273 (PMC11783541; doi:10.1021/acsami.4c17273)
Supplement: Supplementary file 1 — am4c17273_si_001.pdf [file am4c17273_si_001.pdf]

Supporting Information

Highly efficient, electro-thermal heater based on Marangoni-driven, oriented rGO/ PEI nanolaminates

Christos Pavlou[1,3#], Nikolaos Koutroumanis[1,4#], Anastasios C. Manikas[1,2], Maria Giovanna Pastore Carbone[1], George Paterakis[1], Costas Galiotis[1,2*]*

[1]Institute of Chemical Engineering Sciences, Foundation of Research and Technology- Hellas (FORTH/ICE-HT), Stadiou Street, Platani, Patras, 26504 Greece.

[2]Department of Chemical Engineering, University of Patras, Patras, 26504 Greece

[3]Delft University of Technology, Department of Microelectronics, Faculty of Electrical Engineering, Mathematics and Computer Science, Delft, 2600 AA, the Netherlands

[4]Application Driven Research & Innovative Engineering (ADRINE), Patras Science Park, Stadiou Street, Platani, Patras, 26504 Greece

E-mail: c.galiotis@iceht.forth.gr, galiotis@chemeng.upatras.gr, a.manikas@iceht.forth.gr

*Corresponding authors #authors contributed equally to this work

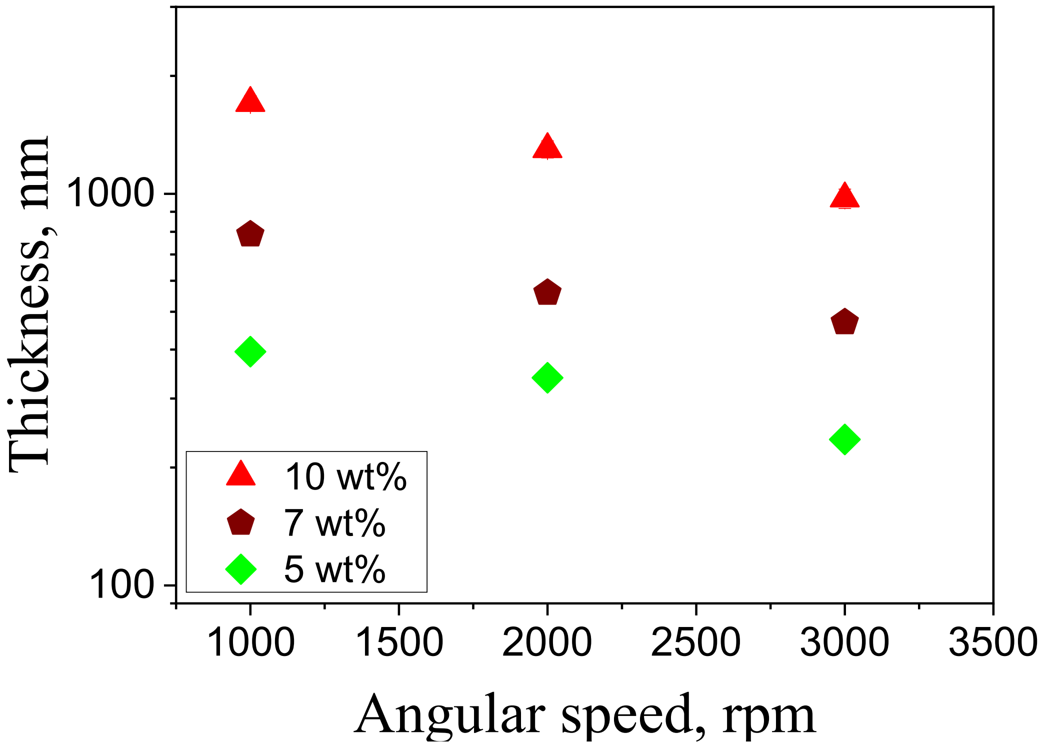

Figure S1: Thickness of PEI nanofilms as a function of angular speed for polymer solutions with different PEI concentrations in cyclohexanone.

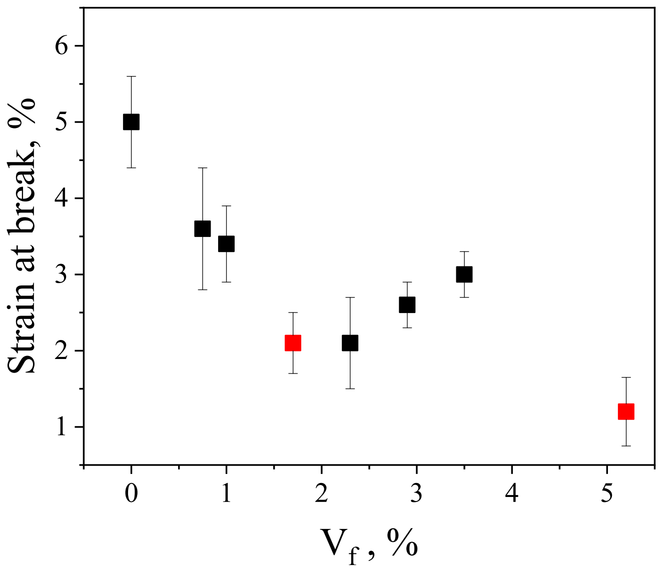

Figure S2: Strain at break values of rGO/PEI nanolaminates for the different volume fractions of rGO.

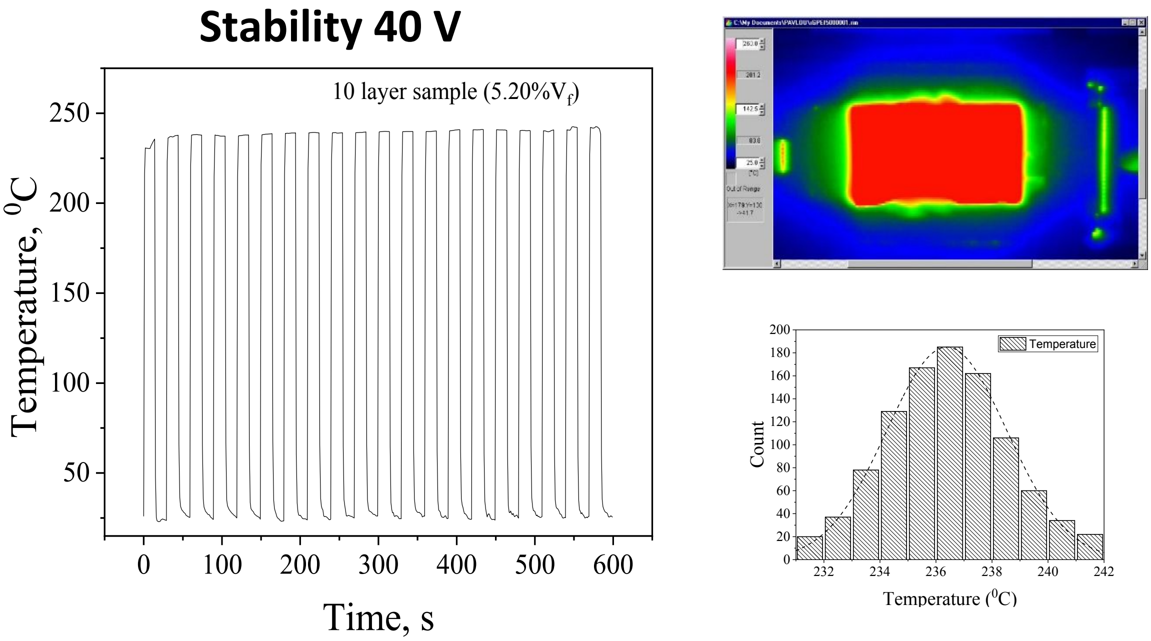

Figure S3: Cyclic test on 10 layers double rGO/PEI nanolaminates and a thermal image of mounted sample at 40 volts with the histogram with the temperature distribution along the sample.

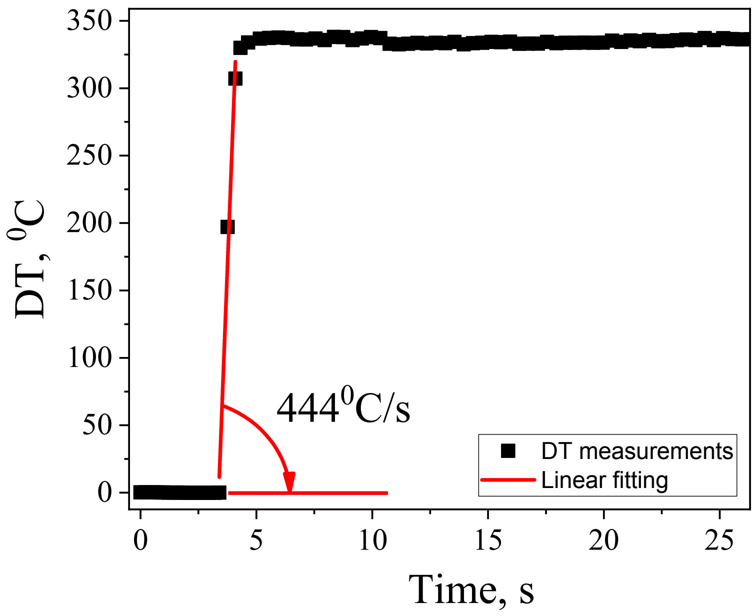

Figure S4: Temperature increment versus time of rGO/PEI nanolaminate with 5.20% volume, for maximum applied voltage.

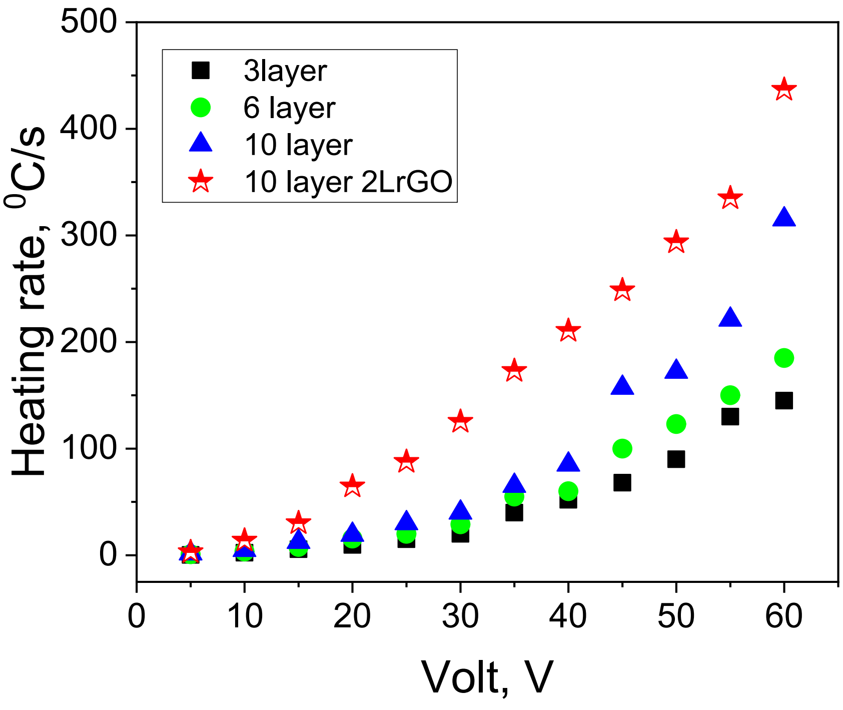

Figure S5: Measured heating rates of rGO/PEI nanolaminates for the different volume fractions of rGO.

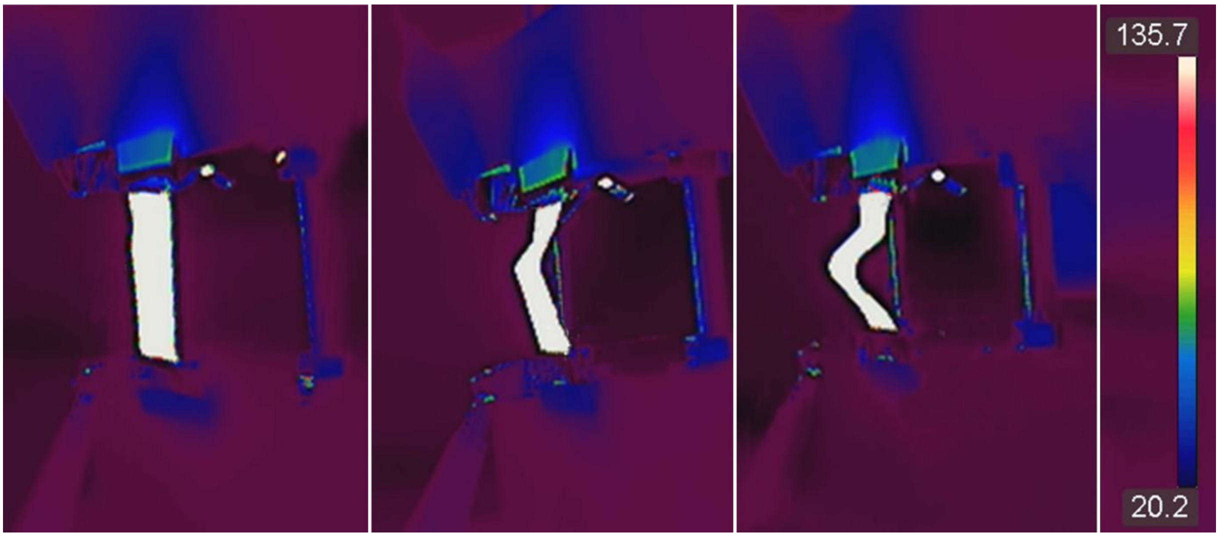

Figure S6: Real-time IR images of the 10-layer nanolaminate with V_f = 5.2 % upon incremental bending. A DC voltage of 25 V is applied to the specimen.

Table S1: Joule heat benchmarking

Type of Heater	Material	Substrate	Heating rate (C/s)	Max. T (C)	Thermal Efficiency C/(W/cm2)	Ref.
Laminate-Composite	PEI-MARANGONI RGO 5.20%vol	PEI	444	360	150	THIS WORK
	3.00%vol		330	240	173	
	1.00%vol		175	180	207	
	0.75%vol		127	145	260	
Laminate-Composite	AgNFs/PtNFs	Silk fibroin	2	160	152	[1]
Composite-multilayer	Ag NWs/ITO-NPs), 20-layer (ITO-NPs/Ag	Norland optical adhesive 63	1.2	120	328	[2]
	3-layer (ITO-NPs/Ag NWs/ITO-NPs NOA film					
Composite	PEDOT:sulf	Glass	1.2	120	~280	[3]
Composite-multilayer	Ni fibers (aligned and non-aligned)	PET/PDMS	N/A	26	73.5	[4]
Laminate	Graphene	Styrene-butadiene rubber	3.3	163	127	[5]
Composite fiber	CNTs	Cotton	2	90	201	[6]
Coating	Ag NW/PEDOT:PSS	Glass	1.1	110	179	[7]
	FTO/AZO	Glass	0.4	70	119	[8]
coating	rGO/CNT/AgNWs	PET	N/A	94.5	25.6	[9]
coating	CNTs	Glass	2	47	137	[10]
textile	Graphene fibers	N/A	571	424	N/A	[11]
coating	AZO/Ag-SnOx/AZO-based TFH	PI	225	438	N/A	[12]
Composite	CNT/PVA	PVA	15	120	N/A	[13]
sheet	CNT	-	3.3	200	N/A	[14]

coating	CVD Graphene 1 layer	PET	>10	80	60	[14]
coating	CVD Graphene 5 layer	PET	>10	85	45	[15]
coating	SWCNT	Glass/PET	2.3	140	N/A	[16]
coating	MWCNT	Glass/PET	4.4	80	N/A	[17]
coating	rGO	PI	16	206	N/A	[18]
coating	Graphene	plastic	0.7	80	N/A	[19]
coating	rGO/Ag nanowire hybrid ITO NPs	quartz	4.5	180	N/A	[20]
coating	Graphene	PET	2	100	N/A	[21]

References

1. Huang, J.; Xu, Z.; Qiu, W.; Chen, F.; Meng, Z.; Hou, C.; Guo, W.; Liu, X. Y., Stretchable and Heat-Resistant Protein-Based Electronic Skin for Human Thermoregulation. *Advanced Functional Materials* **2020,** *30* (13), 1910547.
2. Kim, C.; Lee, M. J.; Hong, S.-J.; Kim, Y.-S.; Lee, J.-Y., A flexible transparent heater with ultrahigh thermal efficiency and fast thermal response speed based on a simple solution-processed indium tin oxide nanoparticles-silver nanowires composite structure on photo-polymeric film. *Composites Science and Technology* **2018,** *157*, 107-118.
3. Gueye, M. N.; Carella, A.; Demadrille, R.; Simonato, J.-P., All-Polymeric Flexible Transparent Heaters. *ACS Applied Materials & Interfaces* **2017,** *9* (32), 27250-27256.
4. Seok Jo, H.; An, S.; Kwon, H.-J.; Yarin, A. L.; Yoon, S. S., Transparent Body-Attachable Multifunctional Pressure, Thermal, and Proximity Sensor and Heater. *Scientific Reports* **2020,** *10* (1), 2701.
5. Wang, F.; Wang, W.; Mu, X.; Mao, J., Anisotropic conductive, tough and stretchable heater based on nacre-like crumpled graphene composite. *Chemical Engineering Journal* **2020,** *395*, 125183.
6. Ilanchezhiyan, P.; Zakirov, A. S.; Kumar, G. M.; Yuldashev, S. U.; Cho, H. D.; Kang, T. W.; Mamadalimov, A. T., Highly efficient CNT functionalized cotton fabrics for flexible/wearable heating applications. *RSC Advances* **2015,** *5* (14), 10697-10702.
7. Ji, S.; He, W.; Wang, K.; Ran, Y.; Ye, C., Thermal Response of Transparent Silver Nanowire/PEDOT:PSS Film Heaters. *Small* **2014,** *10* (23), 4951-4960.
8. Kim, A. Y.; Lee, K.; Park, J. H.; Byun, D.; Lee, J. K., Double-layer effect on electrothermal properties of transparent heaters. *physica status solidi (a)* **2014,** *211* (8), 1923-1927.
9. Kim, C.-L.; Jung, C.-W.; Oh, Y.-J.; Kim, D.-E., A highly flexible transparent conductive electrode based on nanomaterials. *NPG Asia Materials* **2017,** *9* (10), e438-e438.
10. Kang, T. J.; Kim, T.; Seo, S. M.; Park, Y. J.; Kim, Y. H., Thickness-dependent thermal resistance of a transparent glass heater with a single-walled carbon nanotube coating. *Carbon* **2011,** *49* (4), 1087-1093.
11. Wang, R.; Xu, Z.; Zhuang, J.; Liu, Z.; Peng, L.; Li, Z.; Liu, Y.; Gao, W.; Gao, C., Highly Stretchable Graphene Fibers with Ultrafast Electrothermal Response for Low-Voltage Wearable Heaters. *Advanced Electronic Materials* **2017,** *3* (2), 1600425.
12. Wang, Z.; Li, J.; Xu, J.; Huang, J.; Yang, Y.; Tan, R.; Chen, G.; Fang, X.; Zhao, Y.; Song, W., Robust ultrathin and transparent AZO/Ag-SnOx/AZO on polyimide substrate for flexible thin film heater with temperature over 400 °C. *Journal of Materials Science & Technology* **2020,** *48*, 156-162.
13. Zhou, B.; Han, X.; Li, L.; Feng, Y.; Fang, T.; Zheng, G.; Wang, B.; Dai, K.; Liu, C.; Shen, C., Ultrathin, flexible transparent Joule heater with fast response time based on single-walled carbon nanotubes/poly(vinyl alcohol) film. *Composites Science and Technology* **2019,** *183*, 107796.
14. Lee, Y.; Le, V. T.; Kim, J.-G.; Kang, H.; Kim, E. S.; Ahn, S.-E.; Suh, D., Versatile, High-Power, Flexible, Stretchable Carbon Nanotube Sheet Heating Elements Tolerant to Mechanical Damage and Severe Deformation. *Advanced Functional Materials* **2018,** *28* (8), 1706007.

15. Zhang, Y.; Liu, H.; Tan, L.; Zhang, Y.; Jeppson, K.; Wei, B.; Liu, J. Properties of Undoped Few-Layer Graphene-Based Transparent Heaters *Materials* [Online], **2020**.
16. Yoon, Y. H.; Song, J. W.; Kim, D.; Kim, J.; Park, J. K.; Oh, S. K.; Han, C. S., Transparent Film Heater Using Single-Walled Carbon Nanotubes. *Advanced Materials* **2007,** *19* (23), 4284-4287.
17. Wang, J.; Fang, Z.; Zhu, H.; Gao, B.; Garner, S.; Cimo, P.; Barcikowski, Z.; Mignerey, A.; Hu, L., Flexible, transparent, and conductive defrosting glass. *Thin Solid Films* **2014,** *556*, 13-17.
18. Sui, D.; Huang, Y.; Huang, L.; Liang, J.; Ma, Y.; Chen, Y., Flexible and Transparent Electrothermal Film Heaters Based on Graphene Materials. *Small* **2011,** *7* (22), 3186-3192.
19. Lee, B.-J.; Jeong, G.-H., Fabrication of defrost films using graphenes grown by chemical vapor deposition. *Current Applied Physics* **2012,** *12*, S113-S117.
20. Im, K.; Cho, K.; Kwak, K.; Kim, J.; Kim, S., Flexible Transparent Heaters with Heating Films Made of Indium Tin Oxide Nanoparticles. *Journal of Nanoscience and Nanotechnology* **2013,** *13* (5), 3519-3521.
21. Kang, J.; Kim, H.; Kim, K. S.; Lee, S.-K.; Bae, S.; Ahn, J.-H.; Kim, Y.-J.; Choi, J.-B.; Hong, B. H., High-Performance Graphene-Based Transparent Flexible Heaters. *Nano Letters* **2011,** *11* (12), 5154-5158.
